# Neurothecoma in the tibial region^☆^

**DOI:** 10.1016/j.abd.2024.02.009

**Published:** 2024-11-09

**Authors:** Mariana Abdo de Almeida, Neusa Yuriko Sakai Valente, Eduardo César Diniz Macêdo, Bruna Nascimento Arruda Scabello, Patrícia Porto de Oliveira Grossi

**Affiliations:** a Department of Pathological Anatomy, Instituto de Assistência Médica ao Servidor Público Estadual, São Paulo, SP, Brazil; b Department of Dermatology, Hospital do Servidor Público Estadual de São Paulo, São Paulo, SP, Brazil

Dear Editor,

A 35-year-old man reported a lesion on his right leg for four years, showing progressive growth. On examination, he had a normochromic nodule on the anterolateral surface of the right leg, which was painful on deep palpation (Fig. 1). A spindle-shaped excision was performed and the painful lesions of the “ENGLAND” acronym – which encompasses eccrine spiradenoma, neuroma, glomus tumor, leiomyoma, angiolipoma, neurilenoma and dermatofibroma – were considered as diagnostic hypotheses. Macroscopically, an elliptical fragment of skin was received, which measured 1.7 × 1.0 × 0.4 cm and showed a finely reticulated epidermal surface, brown in color, with a raised grainy and firm lesion measuring 0.8 × 0.8 × 0.3 cm, grayish in color. The cut surface of the lesion appeared smooth and whitish, with a small central cystic cavity measuring 0.3 cm in diameter, empty and touching the deep margin. On microscopy, the epidermis appeared rectified (Fig. 2). In the dermis, there was a multilobular proliferation of spindle-shaped or stellate cells, with vesicular nuclei and surrounded by abundant mucin (Fig. 3). Immunohistochemistry was performed with S100 protein (polyclonal; Fig. 4A), which was positive, as well as vimentin (Fig. 4B and 4 C). Ki67 immunostaining indicated a low cell proliferation index (Fig. 4D).

**Figure 1:**
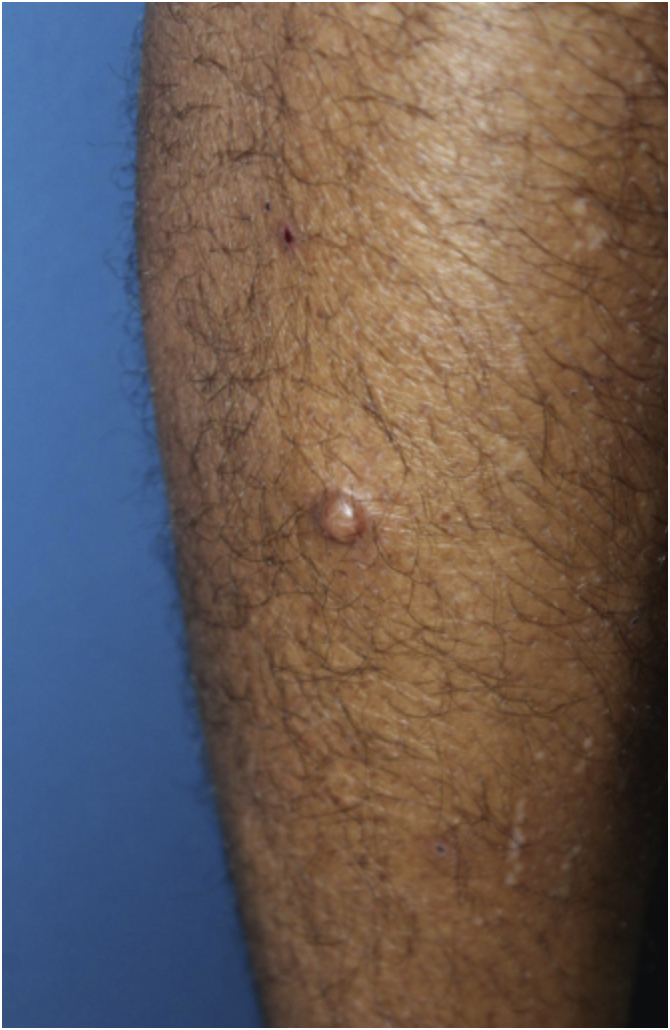
Normochromic papule, with a smooth and regular surface, fibroelastic consistency, located on the anterolateral surface of the right leg.

**Figure 2:**
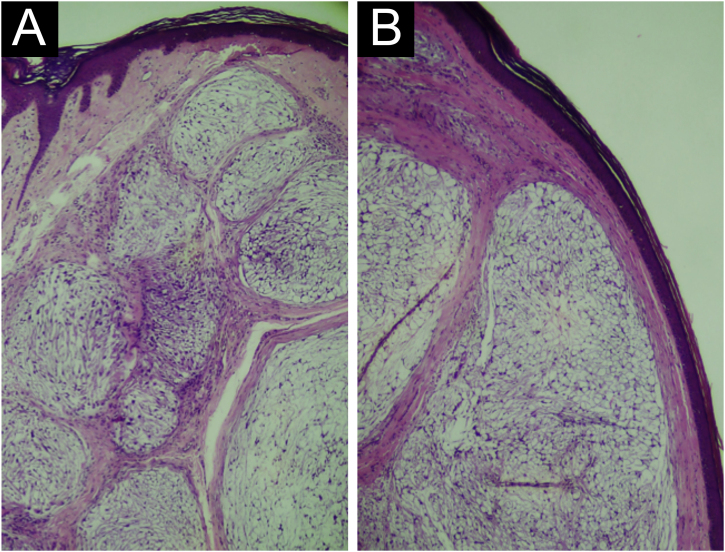
Light Microscopy (A) Rectified epidermis. Dermis with proliferation of spindle-shaped and stellate cells embedded in a myxoid matrix, forming well-circumscribed lobes, separated by fibrous septa (Hematoxylin & eosin, ×40). (B) Proliferation of spindle-shaped and stellate cells, forming well-circumscribed nodules, separated by fibrous septa (Hematoxylin & eosin, ×100).

**Figure 3:**
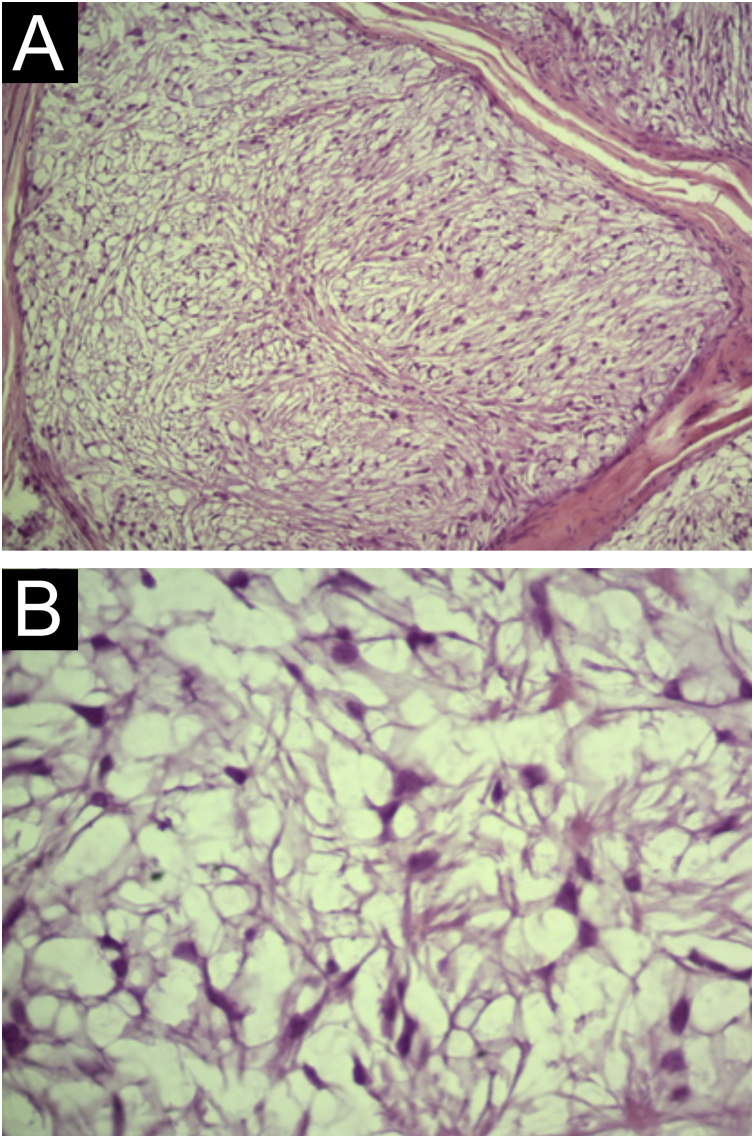
Light Microscopy (A) Cellular atypia or mitoses are not observed (Hematoxylin & eosin, ×100). (B) Detail of stellate and spindle-shaped cells without atypia (Hematoxylin & eosin, ×400).

**Figure 4:**
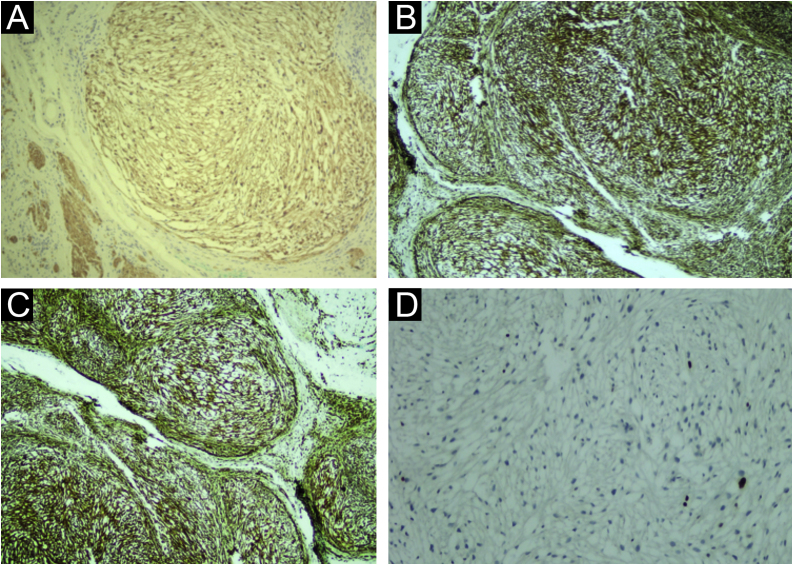
Immunohistochemistry (A) anti-S100 protein antibody positive in the neoplastic cells and in the adjacent nerve, in the left low corner (×100). (B and C) vimentin antibody showing positivity in the neoplastic cells (×100). (D) Ki67 antibody indicating a low cell proliferation index (×100).

Neurothecoma is a rare benign cutaneous tumor derived from the neural sheath described in 1969 by Harkin and Reed as “nerve sheath myxoma”.^1^ Currently, neurothecoma is considered to be derived from the sheath of terminal cutaneous nerves and presents heterogeneous characteristics, due to the fact that neural sheaths consist of cells from different lineages in each of the layers that cover the axons – epineurium, perineurium and endoneurium.^2^, ^3^ Tumors derived from the endoneurium, which consists of abundant longitudinal collagen fibers, glycosaminoglycans, few fibroblasts, Schwann cells and capillaries, present myxoid characteristics and their cells show neuroectodermal and mesenchymal differentiation, and are positive for S100 protein.^4^ Tumors derived from the perineurium and epineurium are more cellular and negative for S100 protein.^3^ Some authors consider the myxoid subtype a degeneration of the cellular variety, which may be supported by the advanced age of patients who present this variant.^5^ On the other hand, other authors consider that these two variants correspond to distinct tumors.^3^

Clinically, they manifest as slow-growing papules, which is why they can take months to years to be diagnosed. They are normally asymptomatic but may be painful.^5^, ^6^ The definitive diagnosis is attained by the histopathological study.^3^ The three morphological patterns have distinct characteristics, but have some similar aspects, such as their location in the dermis and deep tissues, good circumscription, presenting foci of hyalinization, and absence of capsule.^7^ The most common variant is the myxoid (classic variant), which generally occurs in older patients (average age 44.7 years) and is two-fold more common in women. It is characterized by the formation of well-circumscribed lobes of small and thin spindle-shaped and stellate cells, with a vesicular nucleus, without nucleoli, atypia, or mitosis, embedded in an amorphous myxoid matrix. The cellular variant, seen in young patients (average age of 20.5 years) and also more common in women, has a higher cellular density, arranged in clusters or nodules. Generally, the tumor ‒ which is located in the reticular dermis ‒ is separated from the other tissues by a Grenz zone.^5^, ^8^, ^9^ The last histopathological type is the mixed one, which has characteristics of both the myxoid and cellular forms. Neurothecomas can have a large number of differential diagnoses, due to their clinical and histopathological characteristics. Among them are fibrous tumors (dermatofibroma, for instance), histiocytic tumors, lymphocytic tumors, melanocytic tumors, muscle tumors, neural tumors, and vascular tumors. The fact that some neurothecomas clinically present as painful lesions may lead to the diagnostic possibility of one of the painful lesions in the “ENGLAND” acronym. Immunohistochemistry also helps in the diagnosis, mainly with S100 protein, which, when positive, as in the present case, confirms the diagnosis of myxoid neurothecoma.^7^, ^9^

## Financial support

None declared.

## Authors' contributions

Mariana Abdo de Almeida: Approval of the final version of the manuscript; design and planning of the study; drafting and editing of the manuscript; collection, analysis and interpretation of data; effective participation in research orientation; intellectual participation in the propaedeutic and/or therapeutic conduct of the studied cases; critical review of the literature; critical review of the manuscript.

Neusa Yuriko Sakai Valente: Approval of the final version of the manuscript; design and planning of the study; drafting and editing of the manuscript; collection, analysis and interpretation of data; effective participation in research orientation; intellectual participation in the propaedeutic and/or therapeutic conduct of the studied cases; critical review of the literature; critical review of the manuscript.

Eduardo César Diniz Macêdo: Approval of the final version of the manuscript; design and planning of the study; drafting and editing of the manuscript; collection, analysis and interpretation of data; intellectual participation in the propaedeutic and/or therapeutic conduct of the studied cases; critical review of the literature; critical review of the manuscript.

Bruna Nascimento Arruda Scabello: Approval of the final version of the manuscript; drafting and editing of the manuscript; collection, analysis and interpretation of data; intellectual participation in the propaedeutic and/or therapeutic conduct of the studied cases; critical review of the literature; critical review of the manuscript.

Patrícia Porto de Oliveira Grossi: Approval of the final version of the manuscript; drafting and editing of the manuscript; collection, analysis and interpretation of data; intellectual participation in the propaedeutic and/or therapeutic conduct of the studied cases; critical review of the literature; critical review of the manuscript.

## Conflicts of interest

None declared.

## References

[1] Wilson A.D., Rigby H., Orlando A. (2008). Atypical cellular neurothekeoma ‒ a diagnosis to be aware of. J Plast Reconstr Aesthet Surg..

[2] Ramos G.J.A., Torres G.S., Maya A.S.E., Domínguez S.M.Á, De la Torre G.M.E. (2017). Neurotecoma mixoide. Rev Cent Dermatol Pascua..

[3] Papadopoulos E.J., Cohen P.R., Hebert A.A. (2004). Neurothekeoma: report of a case in an infant and review of the literature. J Am Acad Dermatol..

[4] Fullen D.R., Lowe L., Su L.D. (2003). Antibody to S100a6 protein is a sensitive immunohistochemical marker for neurothekeoma. J Cutan Pathol..

[5] Hornick J.L., Fletcher C.D. (2007). Cellular neurothekeoma: detailed characterization in a series of 133 cases. Am J Surg Pathol..

[6] Fetsch J.F., Laskin W.B., Hallman J.R., Lupton G.P., Miettinen M. (2007). Neurothekeoma: an analysis of 178 tumors with detailed immunohistochemical data and long-term patient follow-up information. Am J Surg Pathol..

[7] Levin K.A., Hayden R., Hanau C.A., Galindo L.M. (2002). Cytologic findings of myxoid neurothekeoma: case report based on fine-needle aspiration cytology, immunohistochemistry, and correlating histopathology. Diagn Cytopathol..

[8] Bhatia S., Chu P., Weinberg J.M. (2003). Atypical cellular neurothekeoma. Dermatol Surg..

[9] Sachdev R., Sundram U.N. (2006). Frequent positive staining with NKI/C3 in normal and neoplastic tissues limits its usefulness in the diagnosis of cellular neurothekeoma. Am J Clin Pathol..

